# Inhibition of NOX4-Mediated ROS Production Contributes to Selenomethionine’s Anti-Inflammatory Effect in LPS-Stimulated Bovine Endometrial Epithelial Cells

**DOI:** 10.3390/vetsci12090789

**Published:** 2025-08-22

**Authors:** Luying Cui, Wanting Li, Sasa He, Long Guo, Kangjun Liu, Junsheng Dong, Jianji Li, Heng Wang

**Affiliations:** 1 College of Veterinary Medicine, Jiangsu Co-Innovation Center for Prevention and Control of Important Animal Infectious Disease and Zoonoses, Yangzhou University, Yangzhou 225009, China; mz120221595@stu.yzu.edu.cn (W.L.); mz120231724@stu.yzu.edu.cn (S.H.); yzdxgl@yzu.edu.cn (L.G.); kangjunliu@yzu.edu.cn (K.L.); junsheng@yzu.edu.cn (J.D.); jjli@yzu.edu.cn (J.L.); 2 International Research Laboratory of Prevention and Control of Important Animal Infectious Diseases and Zoonotic Diseases of Jiangsu Higher Education Institutions, Yangzhou University, Yangzhou 225009, China; 3 Joint International Research Laboratory of Agriculture and Agriproduct Safety of the Ministry of Education, Yangzhou 225009, China

**Keywords:** bovine endometrial epithelial cells, NADPH oxidase, NF-κB, inflammation, selenium

## Abstract

Bovine endometritis can be caused by *Escherichia coli* (*E. coli*), from which the lipopolysaccharide (LPS) triggers TLR4/NF-κB-mediated inflammation and reactive oxygen species (ROS) overproduction, resulting in impaired reproductive performance. While NADPH oxidase (NOX) is a critical source of ROS generation, its role in bovine endometrial epithelial cells (BEEC) and modulation by selenium remains unexplored. In this study, primary BEEC was challenged by LPS to assess NOX2/4 expression kinetics. Inhibitors of NOX and NF-κB were applied to observe the role of NOX-derived ROS in BEEC inflammation and in selenomethionine (SeMet)-modulated anti-inflammation. ROS levels were measured by flow cytometry. The changes in inflammatory cytokines, and the proteins related to NOX4 and NF-κB, were analyzed via qPCR and Western blot. As a result, the inhibition of NOX decreased LPS-induced proinflammatory cytokine expression, ROS accumulation, NOX4 level, and the phosphorylation of NF-κB P65 and IκBα. Conversely, the suppression of NF-κB downregulated the levels of ROS and NOX4. Cotreatment with SeMet and a NOX inhibitor further suppressed the inflammatory response, ROS level, and NF-κB pathway activation compared to individual treatment, but had no additive effect on the NOX4 protein level. In conclusion, the NOX4/ROS/NF-κB axis forms a proinflammatory feedback loop in LPS-stimulated BEEC. SeMet mitigates oxidative stress and inflammation partially through NOX4 inhibition.

## Simple Summary

Postpartum uterine infections, often triggered by *Escherichia coli*-derived lipopolysaccharide (LPS), severely impair dairy cow reproductive performance and cause substantial industry losses. Oxidative stress and inflammation are key drivers of endometrial damage. This study demonstrates that LPS induces a proinflammatory feedback loop in bovine endometrial epithelial cells via the NOX4/ROS/NF-κB axis, where NOX4-derived reactive oxygen species (ROS) activate NF-κB, and activated NF-κB further upregulates NOX4. Selenomethionine (SeMet) alleviates this process by inhibiting NOX4 expression, reducing ROS accumulation, and suppressing NF-κB activation. These findings provide a mechanistic basis for using SeMet as a nutritional intervention against bovine endometritis.

## 1. Introduction

Endometritis is a common inflammatory disease that severely impairs the reproductive performance and milk production of dairy cows, resulting in huge economic losses for the global dairy industry [1]. This condition is often caused by bacterial infections, in which *Escherichia coli* (*E. coli*) is one of the most common pathogens [2]. The virulence factor lipopolysaccharide (LPS) from *E. coli* plays a key role in initiating the inflammatory response. LPS is recognized by Toll-like receptor 4 (TLR4), which recruits the adaptor protein myeloid differentiation factor 88 (MyD88) and activates the nuclear factor kappaB (NF-κB) signaling pathway [3], promoting the transcription of inflammatory cytokines such as tumor necrosis factor α (TNF-α) and interleukin-1 β (IL1β), exacerbating endometrial inflammatory responses and tissue damage [4].

Reactive oxygen species (ROS) play a critical amplifying role in LPS-induced inflammatory damage of the bovine endometrium [5]. As key mediators of inflammation, ROS are primarily generated by mitochondrial respiratory chain complexes and the nicotinamide adenine dinucleotide phosphate (NADPH) oxidase (NOX) family, which comprises seven members (NOX1~5 and dual oxidase 1~2) [6]. In cattle with natural endometritis, elevated NOX activity in uterine neutrophils drives respiratory burst and ROS production essential for pathogen clearance. Clinical and subclinical endometritis in cows are both characterized by heightened NOX-associated responses, including superoxide generation and myeloperoxidase activity [7]. NOX1, NOX2, and NOX4 exhibit functional expression in the bovine reproductive system: NOX1 is upregulated in LPS-challenged mammary epithelial cells, contributing to ROS-mediated inflammatory responses [8]; NOX2 promotes ROS overproduction via a MyD88-dependent pathway in *Staphylococcus aureus* lipoteichoic acid-challenged bovine endometrial cells [9]; and NOX4 is transcriptionally upregulated via TLR4 during LPS-triggered inflammation and oxidative stress in the bovine endometrial cell line [10]. The bidirectional regulatory crosstalk between NF-κB activation and intracellular ROS levels further underscores their interconnected roles in inflammation: activated NF-κB transcriptionally upregulates pro-oxidant genes, such as NOX2, elevating cellular ROS to amplify inflammatory responses, while ROS act as signaling molecules to induce tyrosine phosphorylation of IκBα and modulate NF-κB pathway components [11]. In bovine mastitis models, LPS stimulation concurrently induces ROS release in RAW264.7 cells and activates NOX, suggesting a functional link between NOX-derived ROS (NOX-ROS) and cellular inflammatory responses [12]. Despite these insights, two critical mechanistic questions remain uncharacterized in bovine endometrial epithelial cells: first, whether NOX-ROS directly mediates LPS-induced inflammatory responses; second, whether NOX-ROS crosstalks with NF-κB signaling. 

The periparturient period in dairy cows involves heightened metabolic demands and intensified oxidative stress, leading to negative energy balance and immunosuppression [13,14]. Nutritional intervention with selenium (Se) during this phase enhances antioxidant enzyme activity and improves immune cell function, thereby reducing production-related disease incidences while supporting reproductive health [15,16]. Mechanistically, Se exerts anti-inflammation by inhibiting NF-κB activation [17], while its antioxidant properties involve activating cytoprotective pathways such as nuclear factor erythroid 2-related factor 2 (Nrf2). Our laboratory’s prior studies have further demonstrated that Se protects primary bovine endometrial epithelial cells (BEEC) from LPS-induced inflammation and oxidative damage by suppressing NF-κB and activating Nrf2 [18,19]. Notably, while Se’s modulation of NF-κB and Nrf2 pathways is established, whether it influences the NOX-ROS axis in BEEC remains unclear.

The current study aims to characterize the role of NOX-ROS in LPS-induced inflammatory and oxidative stress responses in BEEC. Using the NOX inhibitor diphenyleneiodonium (DPI) and NF-κB inhibitor pyrrolidine dithiocarbamate (PDTC), we analyzed the following: (1) the contribution of NOX activity to proinflammatory cytokine production and ROS accumulation; (2) the regulatory interplay between NOX and NF-κB signaling. Additionally, we investigated whether selenomethionine (SeMet) alleviates oxidative stress by suppressing NOX-mediated ROS generation, given the uncharacterized role of NOX in Se’s anti-inflammatory mechanism in BEEC.

## 2. Materials and Methods

### 2.1. Experimental Design and Treatment

The dynamic changes in the expression of selected NOX genes were examined in BEEC following LPS stimulation. BEEC was treated with LPS (L2880, Sigma, St. Louis, MO, USA) for 0, 2, 4, 8, and 12 h, after which the relative mRNA abundances of cytochrome B-245 beta chain (*CYBB*) and NADPH Oxidase 4 (*NOX4*) were quantified via quantitative real-time PCR (qPCR). For the experiment, LPS was initially dissolved in basal medium at a stock concentration of 1 mg/mL, aliquoted, and stored at −20 °C. The working concentration of 10 μg/mL was prepared fresh for each treatment.

Next, we evaluated the role of NOX in LPS-induced BEEC inflammatory response by using the NOX inhibitor DPI, and explored the regulatory effect of the NF-κB pathway on NOX-ROS production by using the NF-κB inhibitor PDTC. The group arrangements were as follows: the control group (untreated), the LPS group (LPS only), the LPS + DPI (or LPS + PDTC) group, and the LPS + NAC group. DPI (HY-100965, MCE, Monmouth Junction, NJ, USA) was prepared in DMSO and diluted in basal medium to 1 μM working concentration, while PDTC (HY-18738, MCE, Monmouth Junction, NJ, USA) was dissolved directly in basal medium at 10 μM. N-acetylcysteine (NAC, product number: A0737, Sigma, St. Louis, MO, USA) served as a positive control at 5 mM. Cells were pre-treated with 1 μM DPI [20], 10 μM PDTC [21], or 5 mM N-acetylcysteine [22] for 4 h. Subsequently, they were co-treated with 10 μg/mL of LPS for 8 or 12 h.

We measured intracellular ROS levels and analyzed the expression of NOX components (*CYBB*, *NOX4*, and NOX4), as well as the key protein (phosphorylations of P65 and IκBα) level and downstream cytokine expression (*IL1B*, *TNF*, IL-1β, and TNF-α) of the NF-κB pathway.

Finally, to explore the impact of Se on the NOX/ROS/NF-κB axis, we treated the LPS-stimulated BEEC with selenomethionine (SeMet) and/or DPI. The experiment comprised six treatment groups: the control group, LPS group, LPS + DPI group, LPS + SeMet group, and LPS + DPI + SeMet group. SeMet (S3132, Sigma, St. Louis, MO, USA) was prepared in H_2_O and diluted in basal medium to 4 μM working concentration. Cells were first treated with 4 μM SeMet [23] for 24 h, then exposed to DPI for 4 h, and afterwards co-treated with LPS for 8 or 12 h.

### 2.2. Cell Culture

The BEEC was isolated and cultured following established protocols [24]. Briefly, uterine horns were aseptically collected from multiparous non-lactating cows with no history of uterine disease and immediately transported to the laboratory in an icebox. The uterine surface was disinfected with 75% ethanol, followed by extensive rinsing with sterile phosphate-buffered saline (PBS) containing 50 U/mL penicillin–streptomycin. The uterine horns were then longitudinally dissected into 3–4 cm segments and repeatedly washed with PBS supplemented with bispecific antibodies until the supernatant became clear. The tissue fragments were subsequently digested at 4 °C in DMEM/F12 medium (D8900, Sigma, St. Louis, MO, USA) containing 0.1% pronase (P5147, Sigma, St. Louis, MO, USA) for a duration of 12~18 h. After digestion, the endometrial mucosal layer was aseptically scraped, collected in PBS, and centrifuged at 1200× *g* for 5 min. The resulting pellet was washed three times with PBS to remove residual debris. The purified cells were placed back into complete DMEM/F-12 medium supplemented with 15% fetal bovine serum (S711-050S, Suzhou Shuangru Biotech, Suzhou, China) and 50 U/mL penicillin-streptomycin, then seeded into culture flasks and maintained at 37 °C in a humidified 5% CO_2_ incubator. The medium was replaced every 24~36 h to remove non-adherent cells. Upon reaching 80~90% confluence, the cells were passaged by washing with pre-warmed PBS, followed by detachment using trypsin. Digestion was monitored under a microscope and terminated when approximately 80% of the cells had detached. The cell suspension was then centrifuged, and the pellet was resuspended in fresh medium for subculturing or cryopreservation at −80 °C. Cells from passages 4~6 were used for subsequent experiments.

### 2.3. Cell Viability Detection

Cells were plated into 96-well culture plates, with a density of 1 × 10^3^ cells per well, until reaching 60% confluence. Both blank control and drug treatment groups were established. For drug treatment, cells were incubated with a medium containing graded concentrations of DPI (0, 0.25, 0.5, 1, 2, 4, 8, 16, 32 μM) or PDTC (0, 1.25, 2.5, 5, 10, 20, 40, 80, 160 μM) for 24 h. After treatment, the medium was removed and wells were washed three times with PBS. Subsequently, 10 μL CCK-8 reagent (KTA1020, Abbkine, Wuhan, China) and 100 μL basal medium were added to each well, followed by incubation at 37 °C for 2 h. Absorbance was measured at 450 nm using a BioTek microplate reader (BioTek Instruments, Winooski, VT, USA), with 6–8 parallel samples in a single experiment. Cell viability was calculated according to the manufacturer’s protocol.

### 2.4. Intracellular ROS Detection

The membrane-permeable DCFH-DA probe readily crosses cell membranes and undergoes intracellular hydrolysis to yield DCFH, which is subsequently oxidized by ROS to generate fluorescent DCF. The resulting fluorescence intensity is proportional to intracellular ROS level. The cells were seeded in 6-well plates at a density of 1 × 10^6^ cells per well and cultured to 80% confluency in a 37 °C, 5% CO_2_ incubator. Following experimental grouping, cells were treated with LPS and inhibitors for 8 h prior to staining with the ROS assay kit (S0033S, Beyotime, Shanghai, China), with all procedures performed under light-protected conditions. After EDTA-free trypsinization (3 min), digestion was terminated with complete DMEM/F-12 medium. Cell suspensions were centrifuged at 1200× *g* for 5 min, followed by supernatant removal. The DCFH-DA probe was diluted 1:1000 in basal culture medium, and cells were resuspended in serum-free DMEM/F-12 containing the probe. Following 30 min incubation (37 °C, 5% CO_2_) with intermittent mixing of every 3 min, cells were washed three times with PBS. ROS levels were quantified by flow cytometry (CytoFLEX S, Beckman Coulter, Brea, CA, USA) at 488 nm excitation.

### 2.5. RNA Isolation and RT-qPCR

Cells were washed three times with pre-chilled PBS and lysed in 1 mL TRNzol Universal reagent (R401, Vazyme, Nanjing, China). Total RNA was extracted following the reagent’s instruction. With the use of a spectrophotometer (Thermo Scientific NanoDrop 2000, Waltham, MA, USA), the concentration and purity of the extracted RNA were determined, with 260/280 nm absorbance ratio maintained between 1.8 and 2.0. The cDNA synthesis and quantitative PCR were performed using a reverse transcription reagent (AU341-02) and a qPCR reagent (AQ601-01-V2), respectively. Both reagents were purchased from TransGen Biotech Co., Ltd., in China. Then, the reaction was run on the CFX Connect system (Bio-Rad Laboratories, Hercules, CA, USA) with the volume of the reaction system being 20 μL. All primer sequences are listed in Table 1. Beta-actin expression remained stable across treatment groups and was used as an internal control. Relative gene abundance was calculated using the 2^−ΔΔct^ method.

### 2.6. Western Blot

Following group-specific treatments, cells were washed three times with PBS, and the culture medium was discarded. Cells were then lysed on ice using RIPA lysis buffer (C1053, Applygen, Beijing, China), gently scraped, and collected into centrifuge tubes. After 10 min of low-temperature lysis, samples were centrifuged at 12,000× *g* (4 °C, 10 min), and the supernatant was collected. Protein concentrations were quantified using the BCA assay (p0009, Beyotime, Shanghai, China), and total protein concentrations were normalized across groups by adjusting with additional RIPA buffer. Subsequently, 5× SDS Loading Buffer (P1015, Solarbio, Beijing, China) was added, followed by denaturation at 100 °C for 10 min in a metal bath. Aliquots of the prepared protein samples were stored at −80 °C for future use.

For electrophoresis, SDS-PAGE gels were prepared, and equal amounts of protein samples were loaded. Electrophoresis was performed at 80 V for 30 min (stacking gel) and 120 V for 60–90 min (separation gel). Separated proteins were then transferred to PVDF membranes (IPVH00010, Millipore, Burlington, MA, USA) at 200 mA for 90 min. Membranes were blocked with 5% skim milk in TBST for 1 h at room temperature and washed three times with TBST. Primary antibodies, including NOX4 (A23465, ABclonal, Wuhan, China), IκBα (#4814), p-IκBα (#2859), IL-1β (#63124), TNF-α (#3707; all from Cell Signaling Technology, Danvers, MA, USA), NF-κB P65 (AF5006), and P-NF-κB P65 (AF2006; Affinity Biosciences, Changzhou, China), were applied and incubated overnight at 4 °C. After three 5 min TBST washes, membranes were incubated with a horseradish peroxidase (HRP)-conjugated secondary antibody (AF7021, Affinity Biosciences, Changzhou, China) for 1 h at room temperature. Following three additional TBST washes (10 min each), protein bands were visualized using an ECL substrate and imaged with a chemiluminescence detection system (ChemiScope5300Pro, Clinx Science Instruments, Shanghai, China). Band intensities were measured and quantified by ImageJ 9.0 from National Institutes of Health with GAPDH (AF7021, Affinity Biosciences, Changzhou, China) serving as the loading control. The original WB blots used for analysis can be found in Supplementary Materials.

### 2.7. Statistical Analysis

For data analysis, GraphPad Prism 8.0 software (Dotmatics, Boston, MA, USA) was employed. To assess the normal distribution property of the data, the Kolmogorov–Smirnov approach was utilized. It was found that all data conformed to a normal distribution (*p* > 0.05). For determining statistically significant differences, one-way ANOVA was first applied, and this was then followed by Dunnett’s test. The results were presented in the form of the mean ± standard error of the mean (SEM). When *p* < 0.05, the difference was regarded as statistically significant, while *p* < 0.01 indicated an extremely significant difference. Each experiment was independently repeated three times.

## 3. Results

### 3.1. Changes in NOX Gene Expression in LPS-Stimulated BEEC

**Figure 1:**
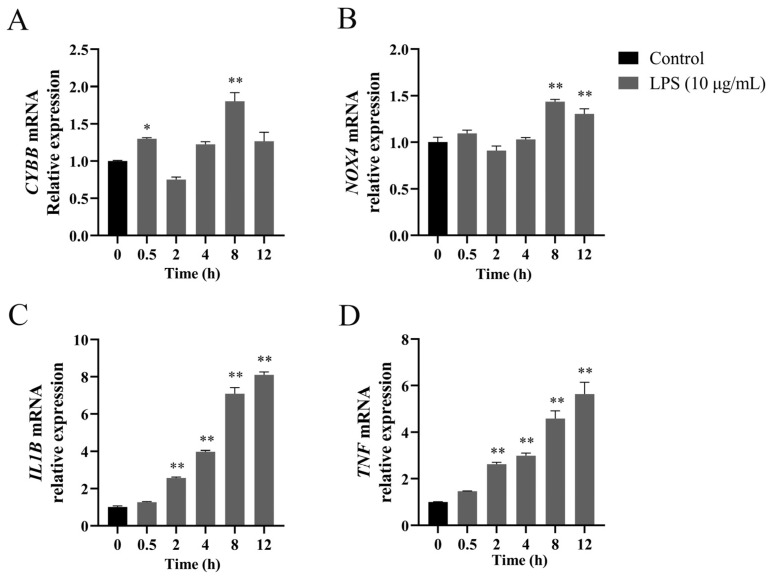
Time-dependent gene expression profiles of NADPH oxidase components and pro-inflammatory cytokines in LPS-treated bovine endometrial epithelial cells. The cells were treated with 10 μg/mL LPS for 0, 2, 4, 8, and 12 h to detect the relative abundance of *CYBB* (**A**), *NOX4* (**B**), *IL1B* (**C**), and *TNF* (**D**) mRNA using quantitative PCR analysis. Gene expression was normalized to the reference gene *β-actin* using the 2^−ΔΔCt^ method. *CYBB*, cytochrome B-245 beta chain. *NOX4*, NADPH oxidase 4. *IL1B*, interleukin 1 beta. LPS, lipopolysaccharide. SEM, standard error of the mean. *TNF*, tumor necrosis factor. Data were expressed as mean ± SEM (*n* = 3). * *p* < 0.05 and ** *p* < 0.01, compared to 0 h.

LPS treatment induced time-dependent upregulation of genes encoding NOX2, NOX4, IL-1β, and TNF-α in BEEC. As shown in Figure 1, *CYBB* mRNA expression increased at 0.5 h (~1.3-fold, *p* < 0.05) and 8 h (~1.8-fold, *p* < 0.01) post-stimulation, and returned to baseline (*p* > 0.05) by 12 h. The *NOX4* transcription demonstrated activation at 8 and 12 h (~1.3 to 1.4-fold, both *p* < 0.01). The relative expression of *IL1B* and *TNF* mRNA exhibited gradual elevation starting at 2 h (*p* < 0.05), and maintained elevation at 12 h (both *p* < 0.01).

### 3.2. DPI Reduced ROS Production and Inhibited NF-κB Activation in LPS-Stimulated BEEC

**Figure 2:**
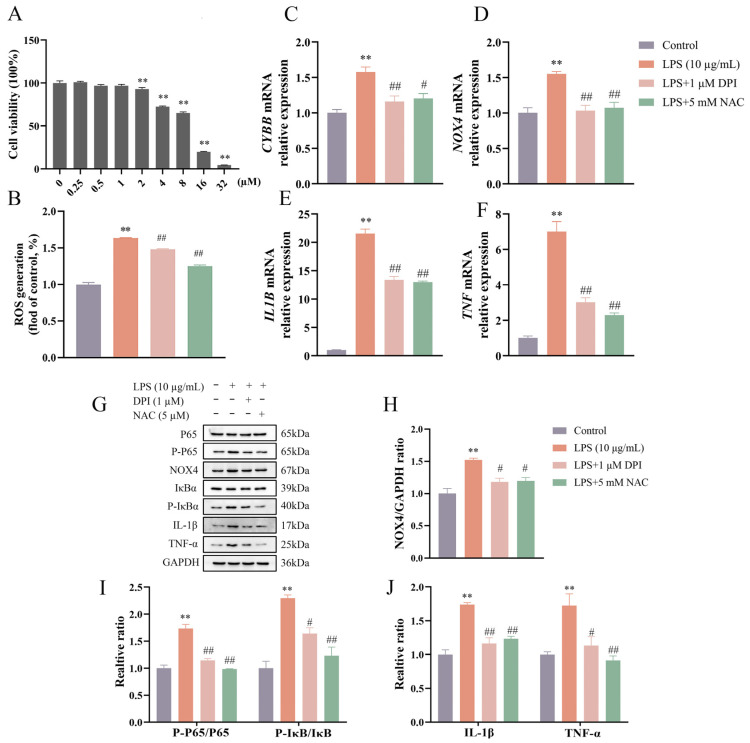
DPI reduced ROS production and inhibited NF-κB activation in LPS-stimulated bovine endometrial epithelial cells. Cell viability was assessed via CCK-8 assay following treatment with various concentrations of DPI for 24 h (**A**). The cells were pretreated with DPI or NAC for 4 h, followed by LPS stimulation to detect the intracellular ROS level by flow cytometry (**B**), the relative mRNA abundance of *CYBB*, *NOX4*, *IL1B*, and *TNF* genes by qPCR (**C**–**F**), and the protein levels of NOX4, P-P65 and P65, P-IκBα and IκBα, IL-1β, and TNF-α by Western blot (**G**–**J**). CYBB, cytochrome B-245 beta chain. DPI, diphenyleneiodonium chloride. NAC, N-acetylcysteine. *NOX4*, NADPH oxidase 4. *IL1B*, interleukin 1 beta. LPS, lipopolysaccharide. ROS, reactive oxygen species. SEM, standard error of the mean. *TNF*, tumor necrosis factor. Data were expressed as mean ± SEM (*n* = 3). ** *p* < 0.01 versus the blank control. ^#^ *p* < 0.05 and ^##^ *p* < 0.01 versus the LPS group.

As shown in Figure 2A,B, the cell viability was not affected (*p* > 0.05) by DPI concentrations up to 1 μM. The DPI concentration of 1 μM was selected for subsequent studies. There was a marked attenuation (all *p* < 0.01) in LPS-induced increases in ROS production, the relative expression of *CYBB*, *NOX4*, *IL1B*, and *TNF* genes, and the protein levels of NOX4, IL-1β, and TNF-α following treatment with DPI or NAC (Figure 2C–J). Specifically, the combined treatment of LPS with NAC/DPI suppressed the expressions of *CYBB* (~1.1-fold vs. control) and *NOX4* (~1.1-fold vs. control) genes and NOX4 protein (~1.2-fold vs. control) to near-control levels. Concurrent with these findings, LPS enhanced the phosphorylation of NF-κB signaling components P65 (~1.6-fold, *p* < 0.01) and IκBα (~2.3-fold, *p* < 0.01), which were both reduced (*p* < 0.01) by pretreatment with DPI or NAC.

### 3.3. PDTC Decreased ROS Production and NOX4 Expression in LPS-Stimulated BEEC

**Figure 3:**
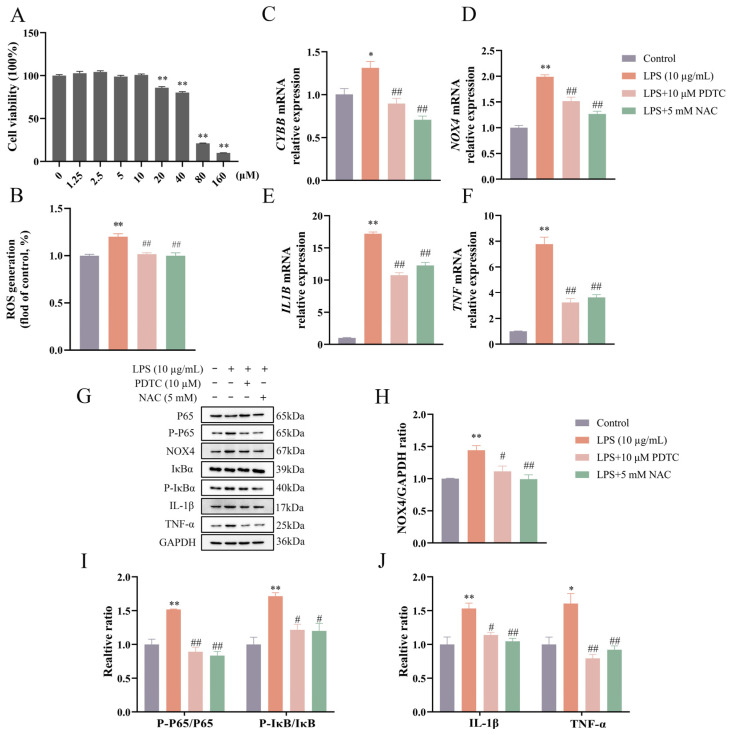
PDTC reduced ROS level and NOX4 expression in LPS-stimulated bovine endometrial epithelial cells. Cell viability was assessed by CCK-8 assay following treatment with various concentrations of PDTC for 24 h (**A**). The cells were pretreated with PDTC or NAC for 4 h, followed by LPS stimulation to detect intracellular ROS level by flow cytometry (**B**), the relative mRNA abundance of *CYBB*, *NOX4*, *IL1B*, and *TNF* genes by qPCR (**C**–**F**), and the protein levels of NOX4, P-P65 and P65, P-IκBα and IκBα, IL-1β, and TNF-α by Western blot (**G**–**J**). *CYBB*, cytochrome B-245 beta chain. PDTC, pyrrolidinedithiocarbamate ammonium. NAC, N-acetylcysteine. *NOX4*, NADPH oxidase 4. *IL1B*, interleukin 1 beta. LPS, lipopolysaccharide. ROS, reactive oxygen species. SEM, standard error of the mean. *TNF*, tumor necrosis factor. Data were expressed as mean ± SEM (*n* = 3). * *p* < 0.05 and ** *p* < 0.01 versus the blank control. ^#^ *p* < 0.05 and ^##^ *p* < 0.01 versus the LPS group.

PDTC concentrations ranging from 0 to 10 μM had no effect (*p* > 0.05) on cell viability (Figure 3A). The concentration of 10 μM PDTC was selected for subsequent experiments. As shown in Figure 3B–J, pretreatment with PDTC led to reductions (*p* < 0.05) in LPS-induced ROS production and proinflammatory cytokine expression. The ratios of P-P65/P65 and P-IκBα/IκBα were decreased (*p* < 0.01) by PTDC under LPS treatment. The expression of *CYBB* and *NOX4* genes (both *p* < 0.01) and NOX4 protein (*p* < 0.05) was decreased in the LPS + PDTC group compared to the LPS group.

### 3.4. NOX Suppression Amplified the Inhibitory Effect of SeMet on ROS Generation and Inflammation in LPS-Stimulated BEEC

**Figure 4:**
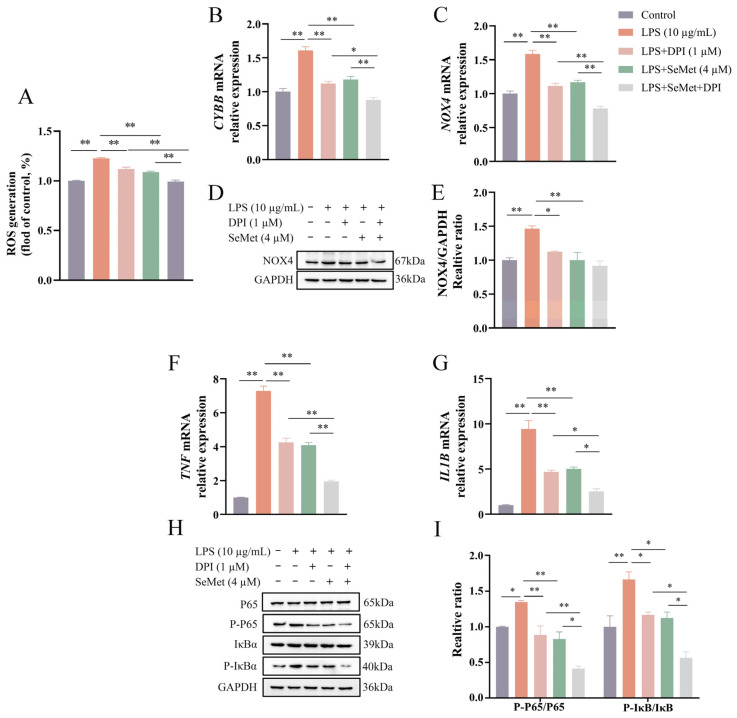
Effect of DPI on SeMet-mediated inhibition of LPS-induced ROS production, NOX expression, and NF-κB signaling in BEEC. Cells underwent pretreatment with SeMet for 24 h and/or DPI for 4 h, followed by LPS stimulation to detect the intracellular ROS level (**A**), the relative expression of *CYBB* (**B**) and *NOX4* (**C**) genes, the NOX4 protein level (**D**,**E**), the relative expression of *TNF* (**F**) and *IL1B* (**G**) genes, and the key protein levels of NF-κB signaling (**H**,**I**). BEEC, bovine endometrial epithelial cells. *CYBB*, cytochrome B-245 beta chain. DPI, diphenyleneiodonium chloride. *NOX4*, NADPH oxidase 4. *IL1B*, interleukin 1 beta. LPS, lipopolysaccharide. ROS, reactive oxygen species. SEM, standard error of the mean. SeMet, selenomethionine. *TNF*, tumor necrosis factor. Data were expressed as mean ± SEM (*n* = 3). * *p* < 0.05 and ** *p* < 0.01.

As shown in Figure 4A–E, both SeMet and DPI attenuated (*p* < 0.01) LPS-induced ROS generation and the mRNA expression of *CYBB* and *NOX4*, with their combined treatment leading to a further reduction (*p* < 0.05) compared to either treatment alone. However, at the protein level, NOX4 expression did not differ (*p* > 0.05) between the LPS + DPI + SeMet group and the LPS + SeMet or LPS + DPI groups. Consistent with the ROS changes (Figure 4F–I), SeMet or DPI alone decreased (*p* < 0.05) the relative expression of proinflammatory cytokines as well as the ratios of P-P65/P65 and P-IκBα/IκBα, while the combination of SeMet and DPI exerted a more pronounced inhibition (*p* < 0.05) in LPS-stimulated BEEC.

## 4. Discussion

*E. coli* is among the most frequently isolated pathogenic bacteria in the bovine uterine cavity [25]. LPS from *E. coli* activates the TLR4/NF-κB signaling cascade and triggers proinflammatory cytokine release in the bovine endometrium [26]. This leads to phosphorylation of the key protein NF-κB p65 and its subsequent nuclear translocation, ultimately inducing gene expression of pro-inflammatory cytokines including IL-1β, IL-6, and TNF-α [27]. Notably in this study, the phosphorylation levels of key NF-κB pathway components (P65 and IκBα) were elevated, accompanied by an upregulated expression of IL-1β and TNF-α, suggesting LPS-induced NF-κB activation and inflammatory response in BEEC.

LPS can stimulate ROS generation in multiple cell types, including BEEC [18]. Consistently, we observed an elevated ROS level upon LPS challenge. As a primary source of ROS, the NOX system was the first identified pathway to generate ROS precursor superoxide as a main function rather than a byproduct [28]. LPS has been found to upregulate NOX expression and activity across cell types: enhancing NOX expression in macrophages [29], inducing p67phox translocation (NOX2 activation) in lung endothelia [30], and increasing NOX4 and ROS levels in human lung epithelial cells and mouse lung tissues [31,32]. Our results align with this paradigm: LPS stimulation elevated ROS levels alongside increased expression of NOX2 and NOX4, suggesting activation of these NADPH oxidase isoforms in BEEC. Although both NOX2 and NOX4 are linked to inflammation, prior work has demonstrated that the cytosolic tail of TLR4 directly binds to the COOH-terminal region of NOX4, and this direct interaction drives LPS-induced ROS production and NF-κB activation. Thus, we prioritized the detection of NOX4 protein over other NOX family members.

To investigate the specific contribution of NOX-ROS in LPS-stimulated BEEC inflammation, we employed DPI, a flavoprotein inhibitor that competitively binds to flavin adenine dinucleotide, blocking electron transfer in NOX and causing irreversible enzyme inhibition. DPI-mediated NOX inhibition has been shown to attenuate enzymatic activity in LPS-stimulated RAW 264.7 macrophages [33]. In this study, DPI suppressed *CYBB* expression, as well as the NOX4 expression at both gene and protein levels in LPS-stimulated BEEC, verifying its NOX inhibitory effect. Consistently, DPI treatment reduced mRNA/protein expression of gp91phox (*CYBB* gene product) and ROS generation in brain tissue following LPS stimulation [34]. In gastric cancer cell models, DPI alleviated oxidative stress by inhibiting NOX4 expression and ROS production [35]. The reduction in LPS-induced ROS levels after DPI treatment confirms the involvement of NOX-ROS in BEEC inflammation. However, the more pronounced ROS suppression by the broad-spectrum antioxidant NAC, compared to DPI alone, suggests that NOX activity contributes only partially to total LPS-triggered ROS production. The participation of additional ROS-producing mechanisms, such as the mitochondrial respiratory chain or other enzymatic sources, warrants further mechanistic investigation. Research on pancreatic diseases has shown that NOX inhibitors reduced LPS-stimulated NF-κB P65 phosphorylation and IL-1β secretion in pancreatic stellate cells, thereby inhibiting pancreatic fibrosis [36]. In agreement with these reports, our data demonstrate that DPI attenuated LPS-triggered ROS generation, NF-κB phosphorylation, and subsequent pro-inflammatory cytokine expression, indicating that NOX pathway inhibition downregulates NF-κB signaling.

PDTC is an NF-κB inhibitor that suppresses NF-κB DNA binding and transcriptional activity, thereby downregulating inflammatory cytokines. A previous study has demonstrated PDTC’s ability to reduce the levels of IL-1β and TNF-α, as well as the ratios of P-P65/P65 and P-IκB/IκB in LPS-stimulated bovine rumen epithelial cells [37]. Consistently, our study confirmed that PDTC inhibited LPS-induced phosphorylation of P65 and IκBα and the expression of IL-1β and TNF-α in BEEC, validating its inhibitory effect on NF-κB signaling. PDTC has been reported to decrease NOX4 expression and alleviate alloxan-induced renal injury [38]. According to the current data, PDTC-mediated NF-κB inhibition concurrently downregulated NOX4 expression, indicating the regulatory role of the NF-κB pathway in NOX4 expression. Collectively, our PDTC and DPI intervention experiments reveal a reciprocal regulatory mechanism between NF-κB signaling and NOX4 expression.

The important role of NOX4/ROS/NF-κB signaling has been proposed in disease processes such as cardiac remodeling and inflammation [39], osteoarthritis [40], and chronic stress [41]. However, these studies did not focus on the mutual influence between NOX4 and NF-κB. A study in LPS-stimulated RAW264.7 cells has demonstrated that NOX-ROS activated the NF-κB signaling pathway, which in turn upregulated the expression of NOX and nitric oxide synthase (NOS), promoting further ROS generation. This creates a self-amplifying “positive feedback loop” that exacerbates oxidative stress and inflammation [12]. Nevertheless, in this report, the specific NOX subtype involved was not mentioned. Based on our findings, a regulatory crosstalk between NOX4 and NF-κB may exist in BEEC, whereby NOX4-generated ROS activates NF-κB, and NF-κB reciprocally enhances NOX4 expression. This NOX4/ROS/NF-κB axis likely represents a key mechanism underlying LPS-induced bovine endometrial inflammation. To further elucidate the direct transcriptional regulation between NF-κB and NOX4, chromatin immunoprecipitation assays can be considered in future studies.

Se supplementation primarily comprises organic Se (e.g., SeMet) and inorganic Se (e.g., sodium selenite), among which organic Se has higher bioavailability and has more advantages in elevating blood Se concentration and enhancing immune function [42]. The protective role of Se against ROS is predominantly mediated by selenoproteins, which possess redox activity and catalyze the reduction of hydroperoxides via thiol groups [43]. In bovine endometrial cells, Se supplementation has been shown to upregulate key antioxidant enzymes, including glutathione peroxidase 1, which detoxifies hydrogen peroxide, and glutathione peroxidase 4, which specifically eliminates lipid peroxides [44]. Additionally, Se inhibits ROS production by modulating the inflammatory signaling pathway. For instance, hydroxy-selenomethionine has been demonstrated to exert anti-inflammatory activity by inhibiting NF-κB pathway activation and subsequent pro-inflammatory cytokine secretion, thereby indirectly reducing ROS generation [45]. In our experiment, pretreatment with SeMet attenuated LPS-induced ROS accumulation in BEEC, consistent with the established antioxidant property of Se. However, this observation alone cannot clarify whether the reduced ROS levels were attributed to suppressed ROS generation or enhanced ROS clearance. Our cotreatment experiment provided insights into this question: The additional ROS suppression achieved when SeMet was administered following NOX inhibition suggests that Se’s antioxidant mechanism extends beyond NOX-ROS regulation. This implies the potential involvement of Se in modulating mitochondrial ROS production and/or augmenting cellular ROS scavenging capacity. Conversely, the finding that NOX inhibitors further decreased ROS levels in SeMet-pretreated BEEC indicates that SeMet only partially regulates NOX-mediated ROS generation.

The thioredoxin (TRX) system comprises NADPH, thioredoxin reductase (TrxR), and thioredoxin. As an essential component of the TrxR active centre (Sec^498^), Se is central to the function of the TRX system. Inhibition of the Se-dependent TRX system enhances the intrinsic NADPH oxidase activity of TrxR, leading to increased superoxide anion (O_2_^−^) production [46]. We speculate this elevation of TrxR-mediated oxidative activity may indirectly impinge on NOX4 function, potentially by altering the availability of critical cofactors (e.g., NADPH) for NOX4 or disturbing the local redox microenvironment. Our results demonstrated that SeMet reduced the LPS-induced expression of *CYBB* and *NOX4* genes and NOX4 protein, indicating that Se suppresses NOX4 expression. When BEEC was co-treated with LPS and DPI, the addition of SeMet further reduced *CYBB* and *NOX4* transcript levels, but not NOX4 protein, suggesting that Se had no effect on NOX4 protein in the presence of NOX inhibitors. Conversely, in cells treated with both LPS and SeMet, additional DPI treatment led to a further decline in *CYBB* and *NOX4* mRNA expression, while NOX4 protein levels remained stable. This implies that the NOX inhibitor loses its effect when SeMet is present, indicating that Se inhibits NOX4. Notably, to fully dissect the precise regulatory mode (direct or indirect) of SeMet on NOX4, follow-up investigations could employ approaches like the luciferase reporter gene assay to probe SeMet’s impact on NOX4 promoter activity, which would help clarify transcriptional-level interactions. Together with the ROS data, these results suggested that the NOX4-ROS pathway contributes to, but does not exclusively account for, Se’s antioxidant activity.

Our findings demonstrated that SeMet pretreatment reduced the expression of *IL1B* and *TNF* while suppressing NF-κB activation, further confirming Se’s anti-inflammatory properties, consistent with our previous observations [19,47]. Previous studies have shown that SeMet inhibits NF-κB signaling and downstream inflammation through multiple mechanisms, including restoring autophagic flux (by alleviating autophagosome–lysosome fusion defects), enhancing autophagic activity, suppressing TLR4 expression, and boosting antioxidant capacity (e.g., via increased GSH-Px activity) [23,48]. Notably, when LPS and DPI were co-administered, additional SeMet treatment further suppressed the proinflammatory cytokine expression and NF-κB pathway activity, suggesting that Se’s anti-inflammatory effects extend beyond NOX inhibition and may involve alternative regulatory pathways. Conversely, in the presence of LPS and SeMet, DPI supplementation further suppressed the inflammatory response of BEEC, indicating that SeMet only partially inhibits NOX activity. Given that SeMet downregulated NOX4 at both mRNA and protein levels, we propose that SeMet suppresses BEEC inflammation, at least in part, through NOX4 inhibition. Nevertheless, since DPI is a non-specific NOX inhibitor, future studies should employ NOX isoform-selective inhibitors/agonists to pinpoint the specific NOX subtypes involved in Se’s antioxidant and anti-inflammatory effects. Moreover, pharmacological inhibitors, in general, may exert off-target effects beyond regulating target proteins. Genetic approaches (e.g., siRNA knockdown, CRISPR-based gene editing, or overexpression) could provide deeper mechanistic insights.

## 5. Conclusions

NOX4 and NF-κB may interact positively, with the NOX4/ROS/NF-κB axis contributing to LPS-induced inflammatory response in BEEC. Moreover, Se likely exerts its antioxidant effects in BEEC by suppressing NOX4-mediated ROS generation.

## Figures and Tables

**Table 1:** The list of primer sequences used for amplification of qPCR.

Gene Name	Primer Sequence (5′-3′)	Product Size (bp)	NCBI Number
*CYBB*	F: CTCAGCTACAACATCTGCCTCACT R: CTGTGATTACATCTTTCTCCTCGTCAT	91	NM_174035.4
*NOX4*	F: GAGCAACAAGCCAGTCACCAT R: TTCTTTGACCATTCGGATTTCC	76	NM_001304775.1
*TNF*	F: CCACGTTGTAGCCGACATC R: CCCTGAAGAGGACCTGTGAG	134	NM_173966.3
*IL1B*	F: AGGTCCATACCTGACGGCTA R: TTGGGTGTCTCAGGCATCTC	132	NM_174092.1
*ACTB*	F: CATCACCATCGGCAATGAGC R: AGCACCGTGTTGGCGTAGAG	156	NM_173979.3

## Data Availability

The data presented in this study are available in the article.

## Supplementary Materials

The following supporting information can be downloaded at https://www.mdpi.com/article/10.3390/vetsci12090789/s1, File S1: Original WB blots used for analysis in Figure 2, Figure 3 and Figure 4.

## Abbreviations

The following abbreviations are used in this manuscript:

*E. coli*	*Escherichia coli*
BEEC	bovine endometrial epithelial cells
*CYBB*	cytochrome B-245 beta chain
DPI	diphenyleneiodonium
IL1β/*IL1B*	interleukin 1 beta
LPS	lipopolysaccharide
MyD88	myeloid differentiation factor 88
NAC	N-acetylcysteine
NADPH	nicotinamide adenine dinucleotide phosphate
NF-κB	nuclear factor kappaB
NOS	nitric oxide synthase
NOX	NADPH oxidase
NOX4/*NOX4*	NADPH Oxidase 4
NOX-ROS	NOX-derived ROS
Nrf2	nuclear factor erythroid 2-related factor 2
PDTC	pyrrolidine dithiocarbamate
ROS	Reactive oxygen species
Se	Selenium
SEM	standard error of mean
SeMet	selenomethionine
TLR4	Toll-like receptor 4
*TNF*	tumor necrosis factor
TNF-α	tumor necrosis factor α
TRX	thioredoxin
TrxR	thioredoxin reductase
