# Supplementary material for: Inhibition of NOX4-Mediated ROS Production Contributes to Selenomethionine’s Anti-Inflammatory Effect in LPS-Stimulated Bovine Endometrial Epithelial Cells
Source: Vet Sci. 2025 Aug 22;12(9):789. doi: 10.3390/vetsci12090789 (PMC12474440; doi:10.3390/vetsci12090789)
Supplement: Supplementary file 1 [file vetsci-12-00789-s001.zip › Original WB images for FIG3.pdf]

1. Treatment design: control, LPS, LPS+PDTC, LPS +NAC

Target protein blot	Image	Note
GAPDH	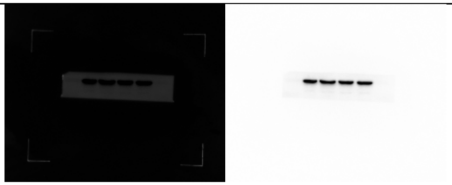	This is replicate 1
NOX4	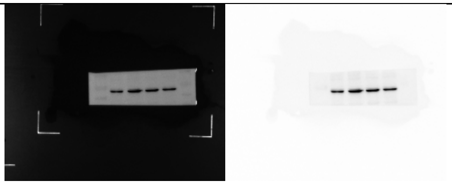	This is replicate 1
GAPDH	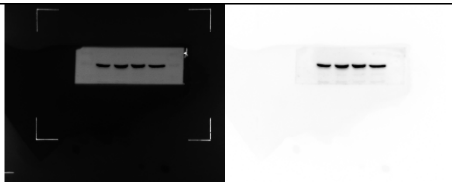	This is replicate 2
NOX4	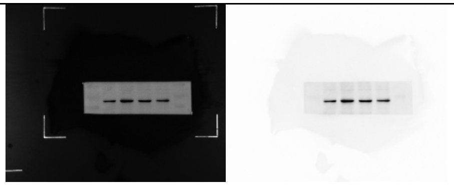	This is replicate 2

<table>
<tr><td>GAPDH</td><td>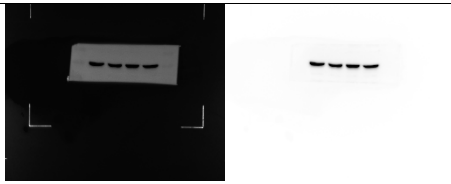</td><td>This is replicate 3</td></tr>
<tr><td>NOX4</td><td>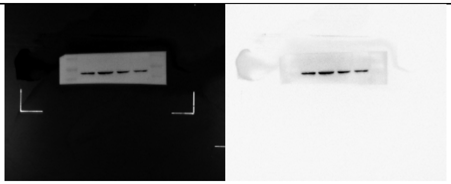</td><td>This is replicate 3</td></tr>
<tr><td>Summary of Triple Bands</td><td>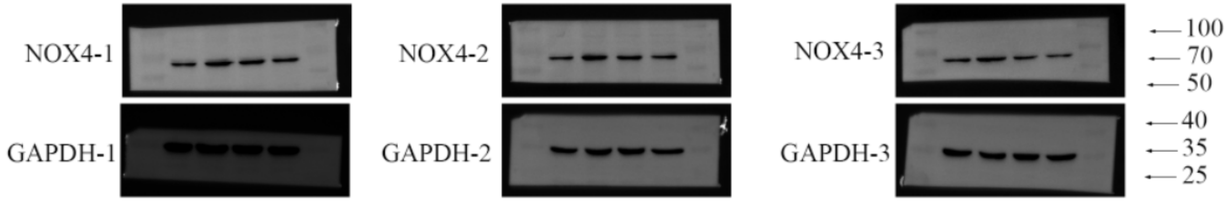
</td><td>NOX4 (67kDa)

GAPDH (36kDa)</td></tr>
</table>

Target protein blot	Image	Note
GAPDH	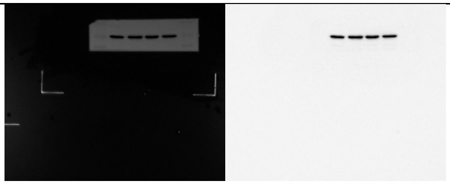	This is replicate 1
P-P65	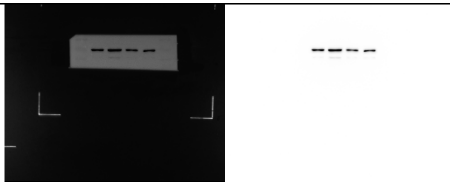	This is replicate 1
GAPDH	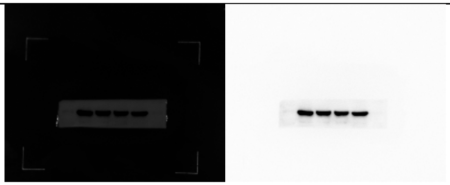	This is replicate 1
P65	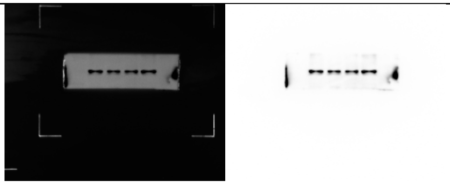	This is replicate 1

GAPDH		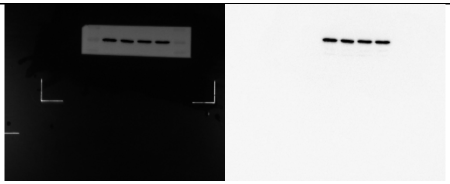	This is replicate 2
PP65		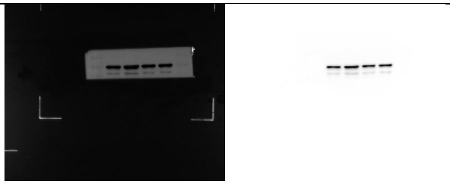	This is replicate 2
GAPDH		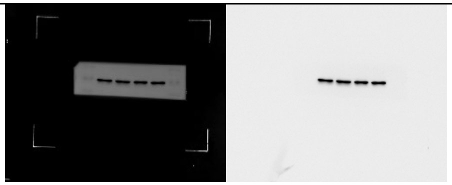	This is replicate 2
P65		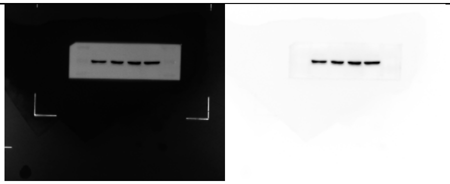	This is replicate 2
GAPDH		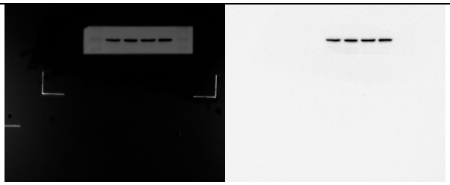	This is replicate 3

PP65	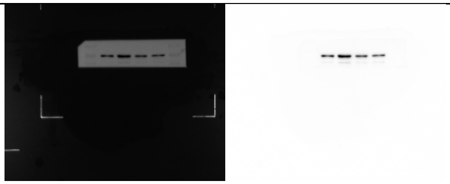	This is replicate 3
GAPDH	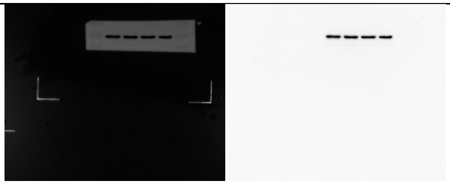	This is replicate 3
P65	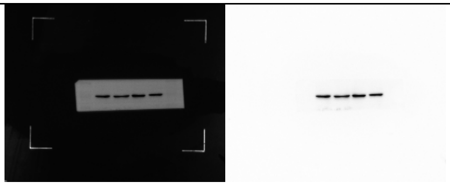	This is replicate 3
Summary of Triple Bands	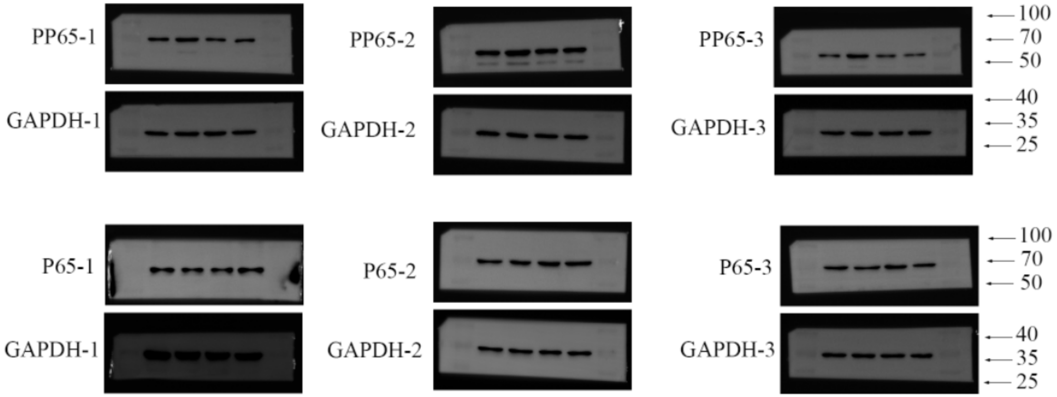	P-P65 (65kDa)<br>P65 (65kDa)<br>GAPDH (36kDa)

Target protein blot	Image	Note
GAPDH	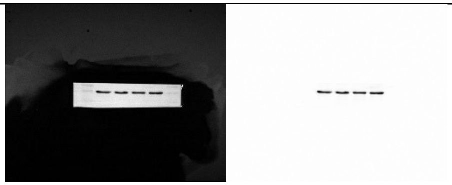	This is replicate 1
P-IκB	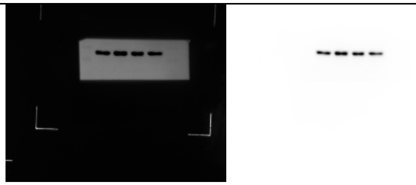	This is replicate 1
IκB	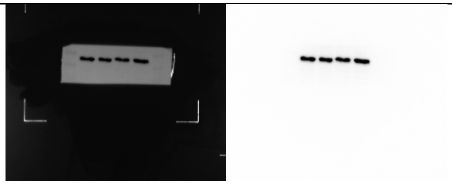	This is replicate 1
GAPDH	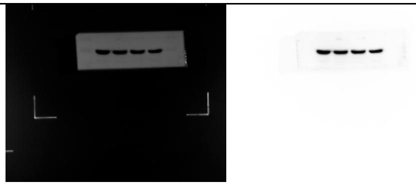	This is replicate 2

P-IκB	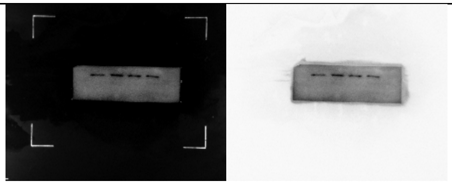	This is replicate 2
IκB	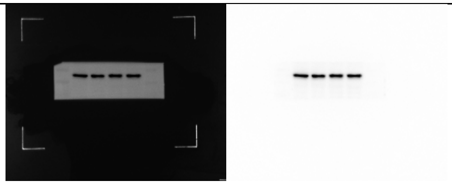	This is replicate 2
GAPDH	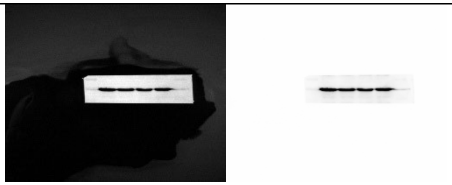	This is replicate 3
P-IκB	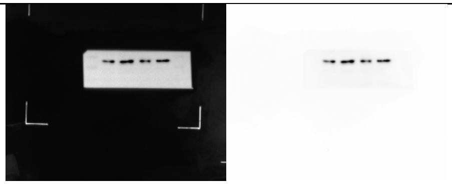	This is replicate 3
IκB	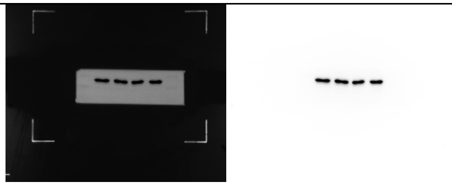	This is replicate 3

Summary of Triple Bands	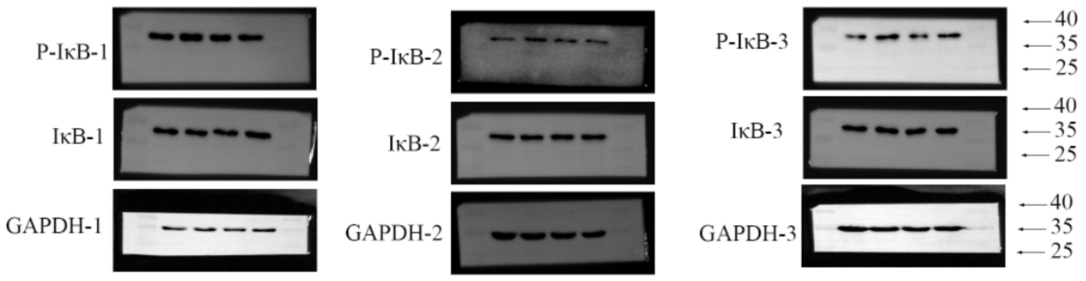	P-IκB (40kDa)<br>IκB (39kDa)<br>GAPDH (36 kDa)

Target protein blot	Image	Note
GAPDH	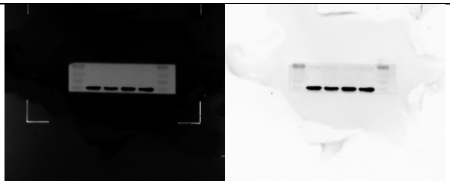	This is replicate1
IL-1β	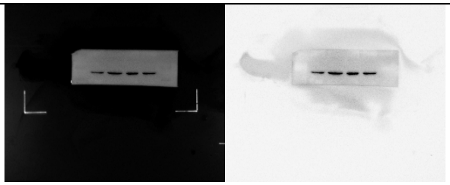	This is replicate 1
GAPDH	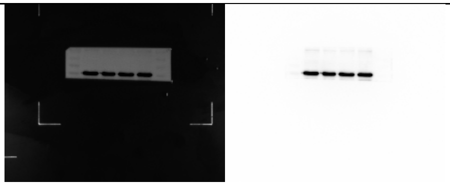	This is replicate 2
IL-1β	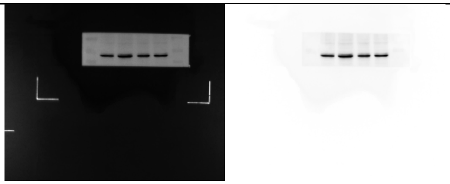	This is replicate 2

GAPDH	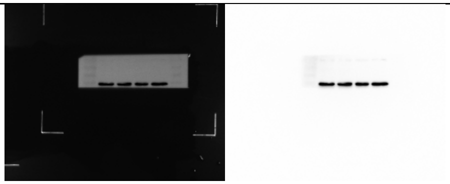	This is replicate 3
IL-1β	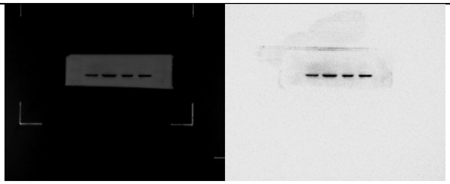	This is replicate 3
Summary of Triple Bands	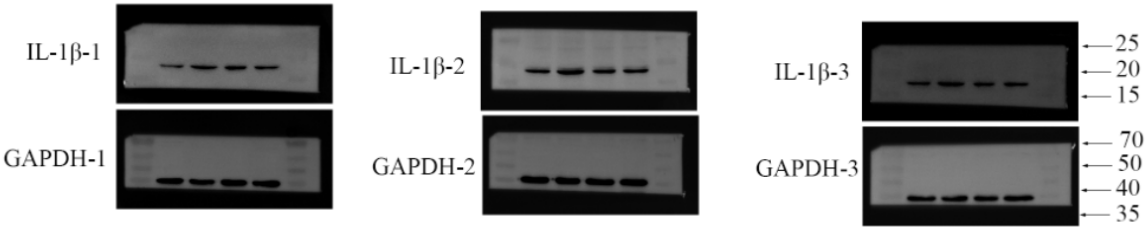	IL-1β (17kDa) GAPDH (36kDa)

Target protein blot	Image	Note
GAPDH	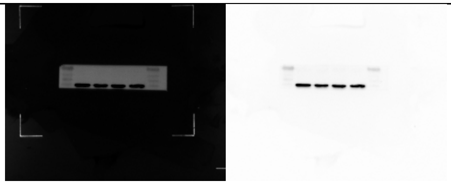	This is replicate 1
TNF-α	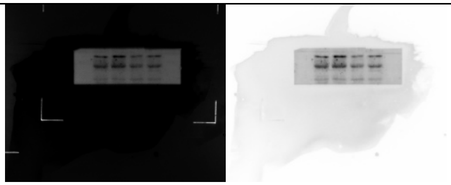	This is replicate 1
GAPDH	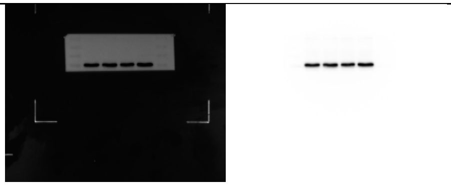	This is replicate 2
TNF-α	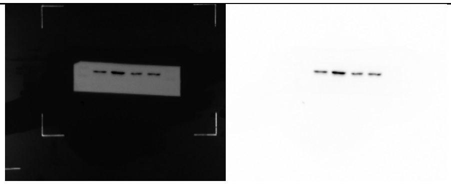	This is replicate 2

GAPDH	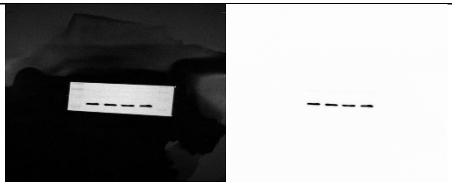	This is replicate 3
TNF-α	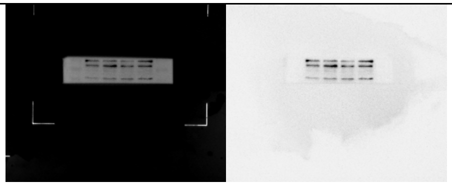	This is replicate 3
Summary of Triple Bands	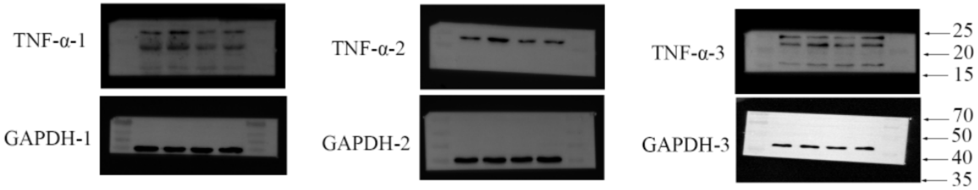	TNF-α (25kDa) GAPDH (36kDa)
