# Supplementary material for: Inhibition of NOX4-Mediated ROS Production Contributes to Selenomethionine’s Anti-Inflammatory Effect in LPS-Stimulated Bovine Endometrial Epithelial Cells
Source: Vet Sci. 2025 Aug 22;12(9):789. doi: 10.3390/vetsci12090789 (PMC12474440; doi:10.3390/vetsci12090789)
Supplement: Supplementary file 1 [file vetsci-12-00789-s001.zip › Original WB images for FIG4.pdf]

1. Treatment design: control, LPS, LPS+DPI, LPS +SeMet, LPS+DPI+SeMet

Target protein blot	Image	Note
GAPDH	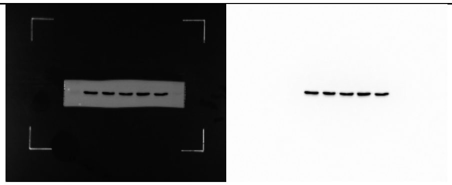	This is replicate 1
NOX4	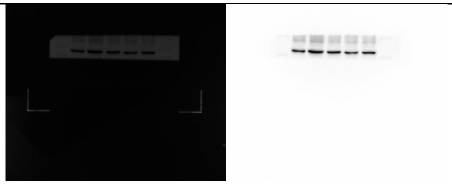	This is replicate 1
GAPDH	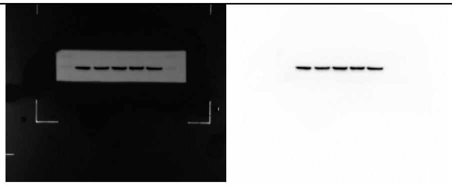	This is replicate 2
NOX4	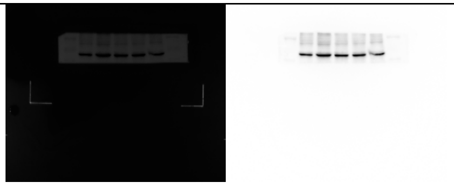	This is replicate 2

GAPDH	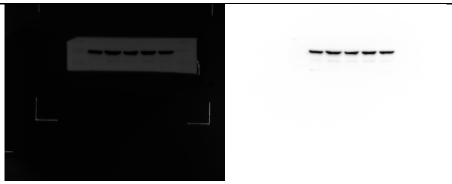	This is replicate 3
NOX4	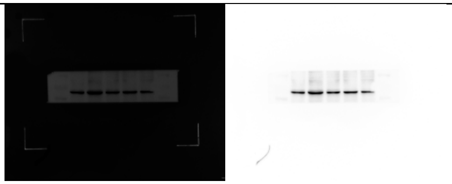	This is replicate 3
Summary of Triple Bands	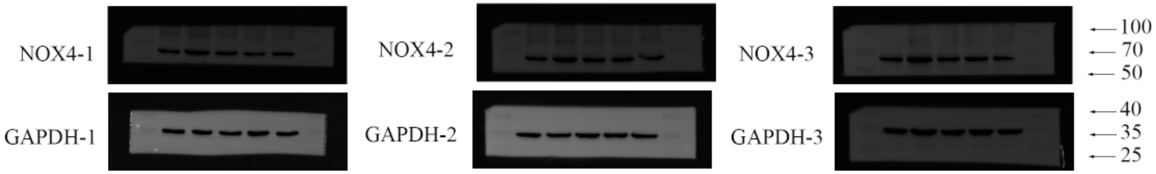	NOX4 (67kDa)<br><br>GAPDH (36kDa)

Target protein blot	Image	Note
GAPDH	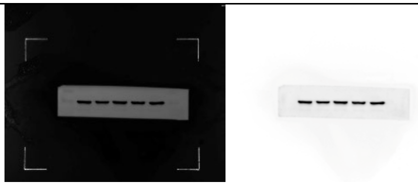	This is replicate 1
P-P65	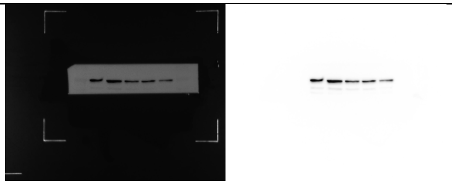	This is replicate 1
GAPDH	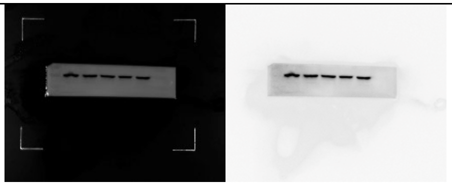	This is replicate 1
P65	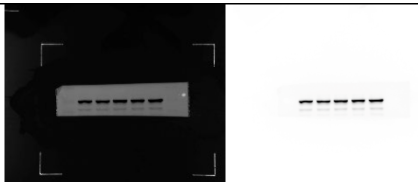	This is replicate 1

GAPDH	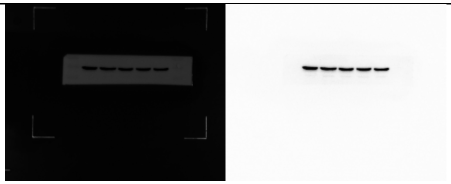	This is replicate 2
PP65	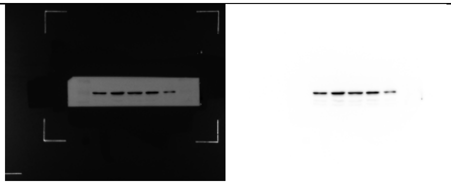	This is replicate 2
GAPDH	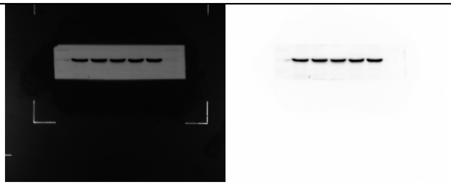	This is replicate 2
P65	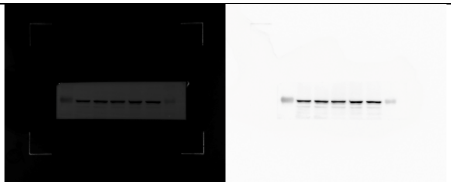	This is replicate 2
GAPDH	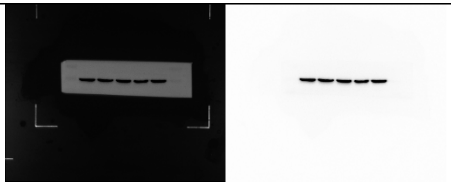	This is replicate 3

PP65	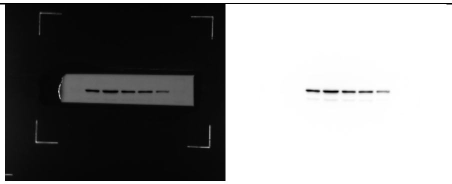	This is replicate 3
GAPDH	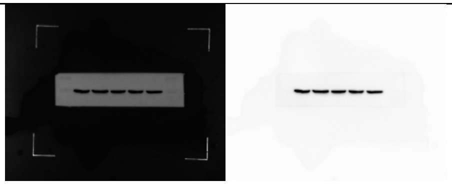	This is replicate 3
P65	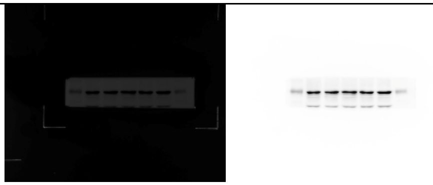	This is replicate 3
Summary of Triple Bands	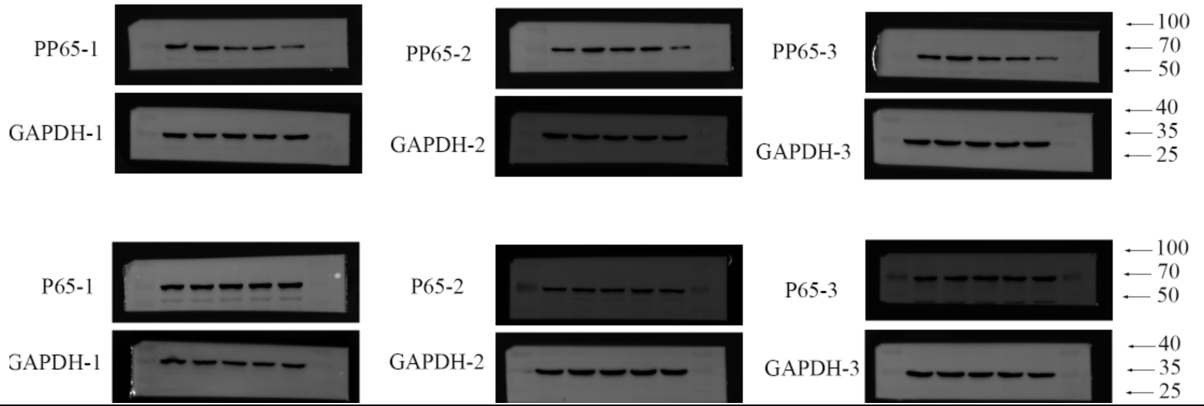	P-P65 (65kDa)<br>P65 (65kDa)<br>GAPDH (36kDa)

Target protein blot	Image	Note
GAPDH	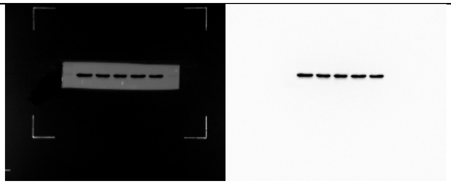	This is replicate 1
P-IκB	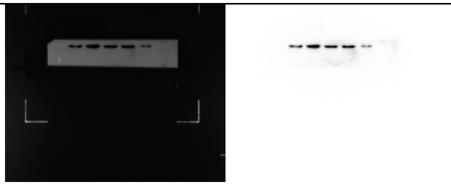	This is replicate 1
IκB	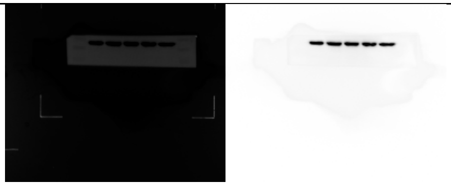	This is replicate 1
GAPDH	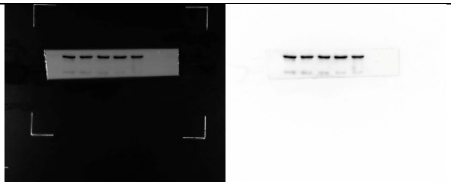	This is replicate 2

P-IκB	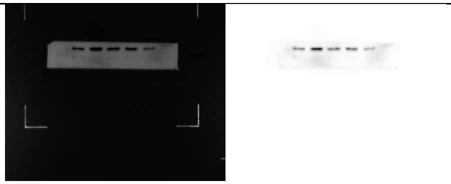	This is replicate 2
IκB	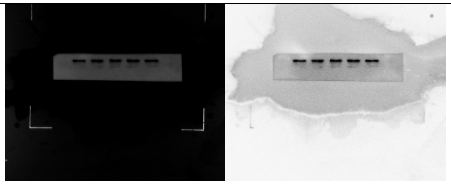	This is replicate 2
GAPDH	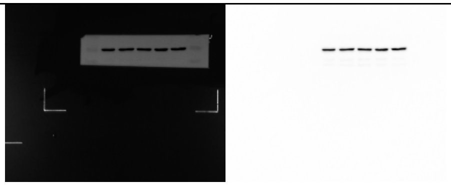	This is replicate 3
P-IκB	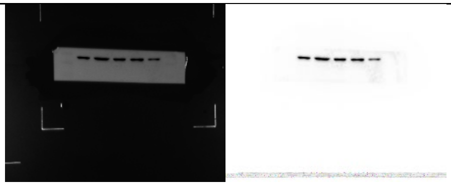	This is replicate 3
IκB	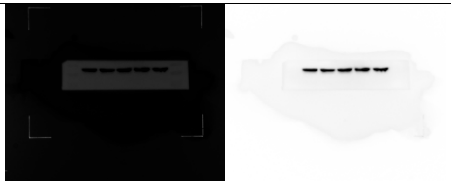	This is replicate 3

Summary of Triple Bands	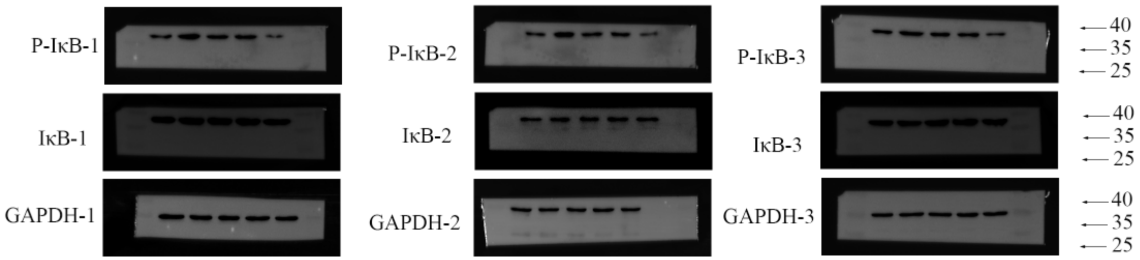	P-IκB (40kDa)<br><br>IκB (39kDa)<br><br>GAPDH (36 kDa)
